# Evaluation of a surgical treatment algorithm for neglected clubfoot in low-resource settings

**DOI:** 10.1007/s00264-021-05058-6

**Published:** 2021-06-19

**Authors:** Manon Pigeolet, Saiful Imam, Gheorghe Cristian Ninulescu, Shafiul Kabir, Pierre R. Smeesters, Hasib Mahmud

**Affiliations:** 1 Faculty of Medicine, Université Libre de Bruxelles, Brussels, Belgium; 2 Impact Foundation Bangladesh, Dhaka, Bangladesh; 3 Department of Orthopedics, Hôpital Universitaire Des Enfants Reine Fabiola, Université Libre de Bruxelles, Brussels, Belgium; 4 Department of Pediatrics, Hôpital Universitaire Des Enfants Reine Fabiola, Université Libre de Bruxelles, Brussels, Belgium; 5 Laboratoire de Génétique Et Physiologie Bactérienne, IBMM, Université Libre de Bruxelles, Brussels, Belgium

**Keywords:** Clubfoot, Developing country, Surgery

## Abstract

**Purpose:**

Idiopathic clubfoot affects approximately 1/1000 alive-born infants, of whom 80–91% are born in low- or middle-income countries (LMICs). This retrospective study aimed to evaluate the morphological, functional, and social outcomes in patients with neglected clubfoot in rural Bangladesh, after receiving surgical treatment.

**Methods:**

Patients received a posteromedial release (PMR) with or without an additional soft tissue intervention (group 1), a PMR with an additional bony intervention (group 2), or a triple arthrodesis (group 3) according to our surgical algorithm. Patients were followed until two year post-intervention. Evaluation was done using a modified International Clubfoot Study Group Outcome evaluation score and the Laaveg-Ponseti score.

**Results:**

Twenty-two patients with 32 neglected clubfeet (ages 2–24 years) received surgical treatment. Nineteen patients with 29 clubfeet attended follow-up. At two year follow-up an excellent, good, or fair Laaveg-Ponseti score was obtained in 81% (group 1), 80% (group 2), and 0% (group 3) of the patients (*p* value 0.0038). Age at intervention is inversely correlated with the Laaveg-Ponseti score at two year follow-up (*p* < 0.0001). All patients attended school or work and were able to wear normal shoes.

**Conclusion:**

Our treatment algorithm is in line with other surgical algorithms used in LMICs. Our data reconfirms that excellent results can be obtained with a PMR regardless of age. Our algorithm follows a pragmatic approach that takes into account the reality on the ground in many LMICs. Good functional outcomes can be achieved with PMR for neglected clubfoot. Further research is needed to investigate the possible role of triple arthrodesis.

**Supplementary Information:**

The online version contains supplementary material available at 10.1007/s00264-021-05058-6.

## Introduction

Idiopathic clubfoot, or congenital talipes equinovarus (CTEV), is the most common musculoskeletal congenital malformation needing intensive orthopedic treatment [1]. Clubfoot affects approximately 1/1000 alive born infants [1, 2], of whom 80–91% are born in low- or middle-income countries (LMICs) [2, 3]. A clubfoot presents with malformation at the bony, tendinous, muscular, and articular level [4, 5]. The foot presents with a midfoot cavus, forefoot adduction, hindfoot varus, and a hindfoot equinus. This gives the typical image of an inward turned foot with the sole of the foot being positioned vertically instead of horizontally [4–7].

The non-operative Ponseti treatment protocol remains the golden standard for clubfoot treatment in low-, middle-, and high-income countries with very good results if treatment is started before walking age [7–9]. However, inability to access proper care remains an issue in LMICs [2, 3, 10]. Only about 15% of affected children are able to access treatment in LMICs [3].

When treatment is not initiated before walking age, the clubfoot becomes a neglected clubfoot [2], which puts the child at risk for developing painful feet, a reduced mobility, and less access to education. It also impacts the broader social context of the child by lowering the standard of living for the entire family and placing a burden on the community in which it lives due to loss of productivity [2, 10, 11]. Neglected clubfoot will often need surgical treatment [2, 9, 12]. The most widely used techniques include an extensive posterior, lateral, and medial soft tissue release, often with navicular, cuboid or first metatarsal osteotomies or a triple arthrodesis [2, 5, 13]. Little research is available on long-term outcomes of surgical treatment for neglected clubfoot in LMICs [2, 12].

In Bangladesh specifically, an estimated 5000 children are born annually with clubfoot [14]. The Bangladeshi national clubfoot program, organized by the non-governmental organization (NGO) “Walk for life,” achieves to provide access to care to approximately 50% of patients across the country [3, 15]. The Impact Foundation Bangladesh is a NGO, established in 1993, providing preventive and curative health care services against avoidable disabilities to women and children through various projects in Bangladesh [16]. Surgical care for neglected clubfoot is provided at their medical centers in cities Chuadanga and Meherpur around 200 km east of the capital Dhaka, in Eastern Bangladesh, and on their floating hospital “Jibon Tari” which visits several rural communities throughout the country every year along the major rivers of Bangladesh.

This retrospective study aimed to evaluate the morphological, functional, and social outcomes in patients with neglected clubfoot in rural Bangladesh, after receiving surgical treatment. We hypothesized that providing surgical treatment for patients with neglected clubfoot ameliorates their foot morphology, lessens their pain, and improves their overall personal and social functioning and wellbeing.

## Materials and methods

We undertook a retrospective cohort study comparing the outcome of 3 different types of surgical treatments for neglected clubfoot in Bangladesh after a surgical campaign in November 2017. Inclusion criteria for receiving surgical treatment were as follows: rigid and non-reducible neglected clubfoot and clubfoot of idiopathic origin. All surgeries have been done by the same surgical team including a Bangladeshi orthopeadic surgeon and two visiting Belgian paediatric orthaopedic surgeons. Exclusion criteria to receive surgical treatment for neglected clubfoot in this program were as follows: underlying neuromuscular disorder, prior failed conservative Ponseti treatment for children under the age of two years and travel time to the hospital of more than four hours.

**Fig. 1:**
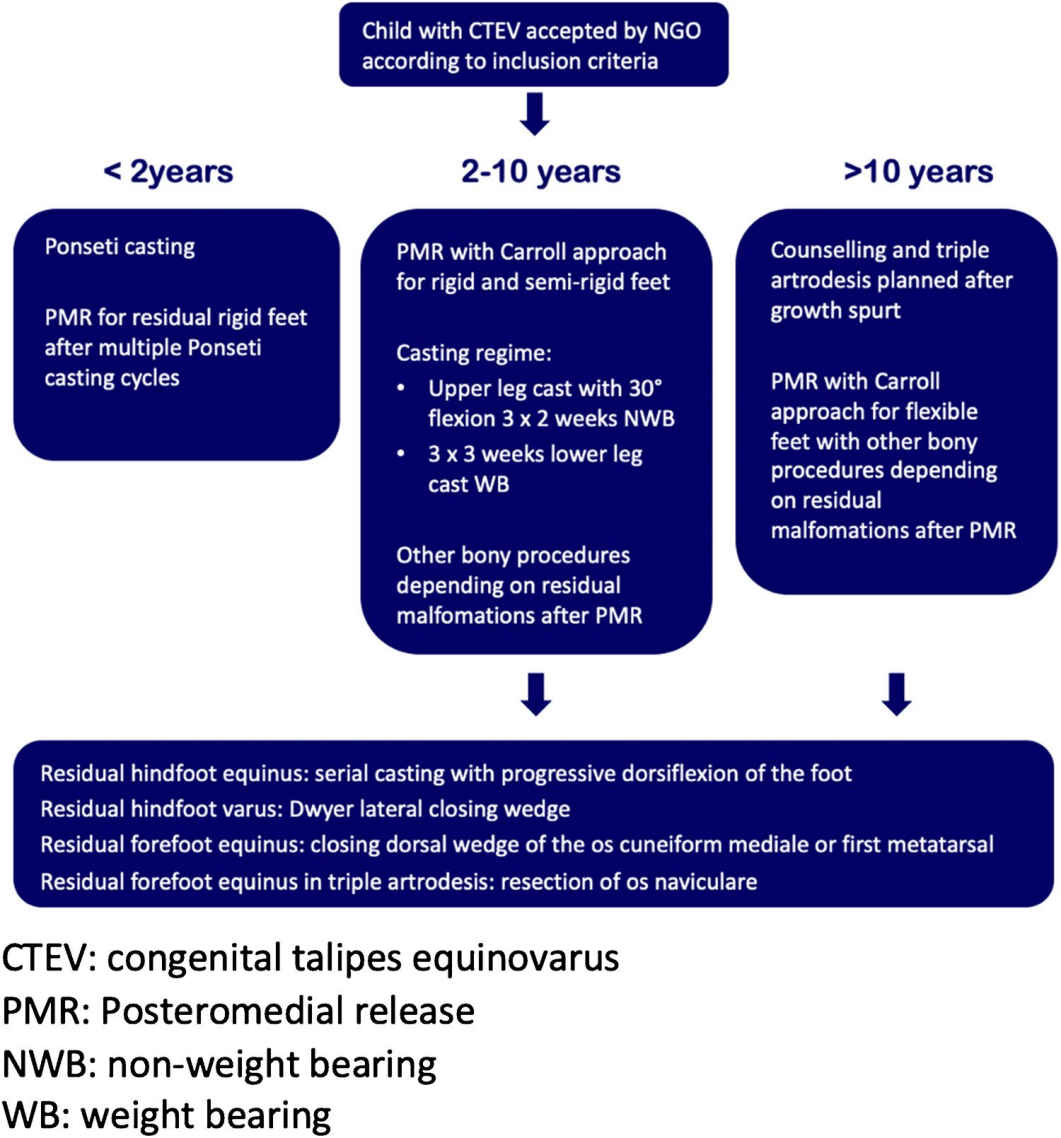
Surgical algorithm

The type of surgical care being delivered was based on an expert algorithm created by the surgical team. Age was used as the major determinant for type of surgery. The algorithm is presented in Fig. 1.

**Photos. 1-3:**
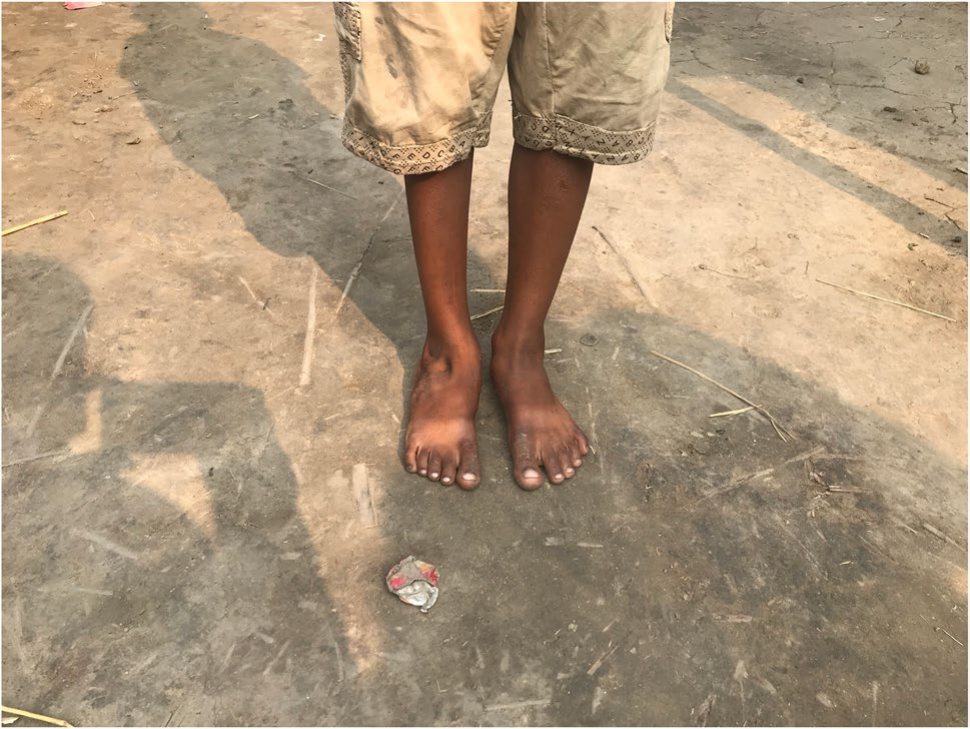

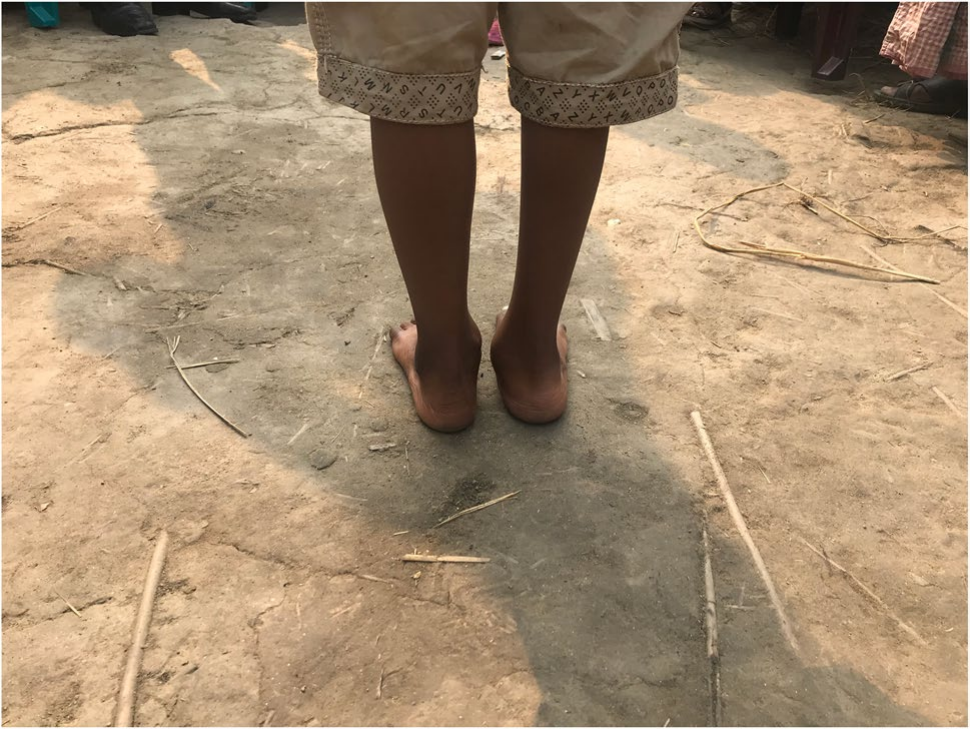

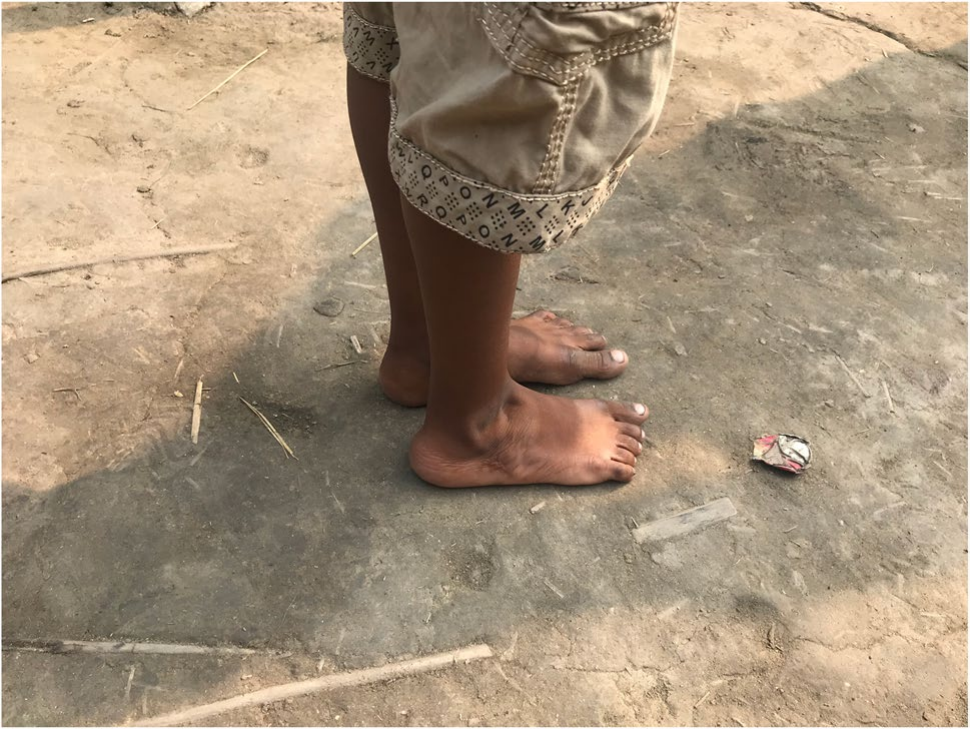
Two-year post-operative status after right PMR with TATT in 5-year-old boy

**Photos. 4-9:**
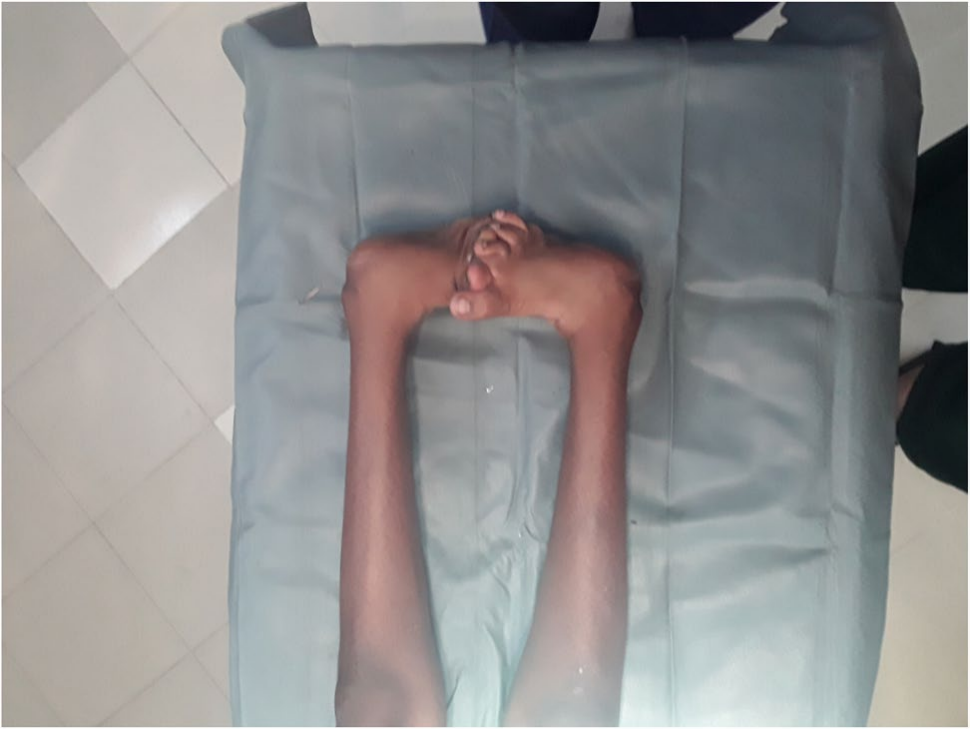

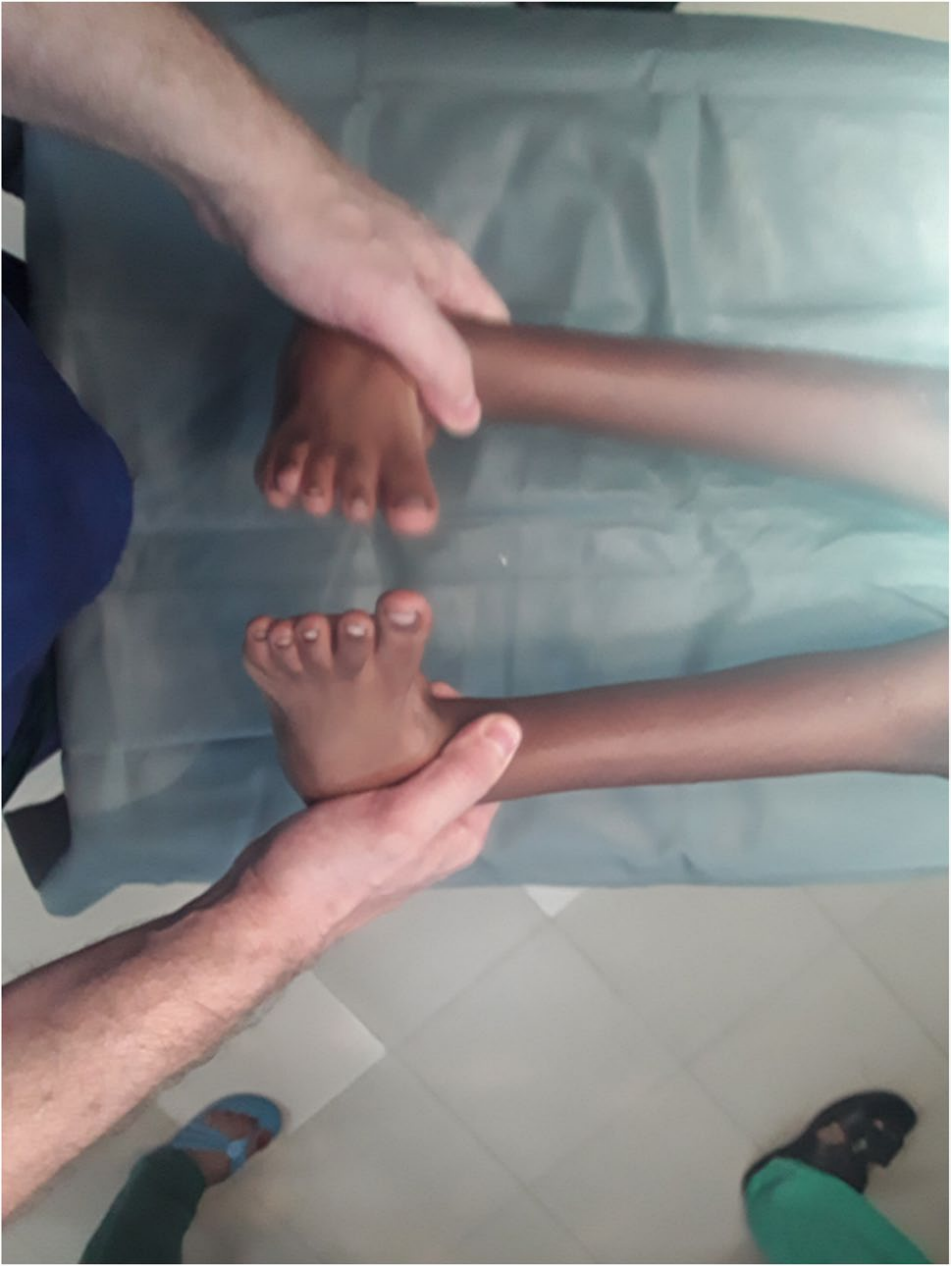

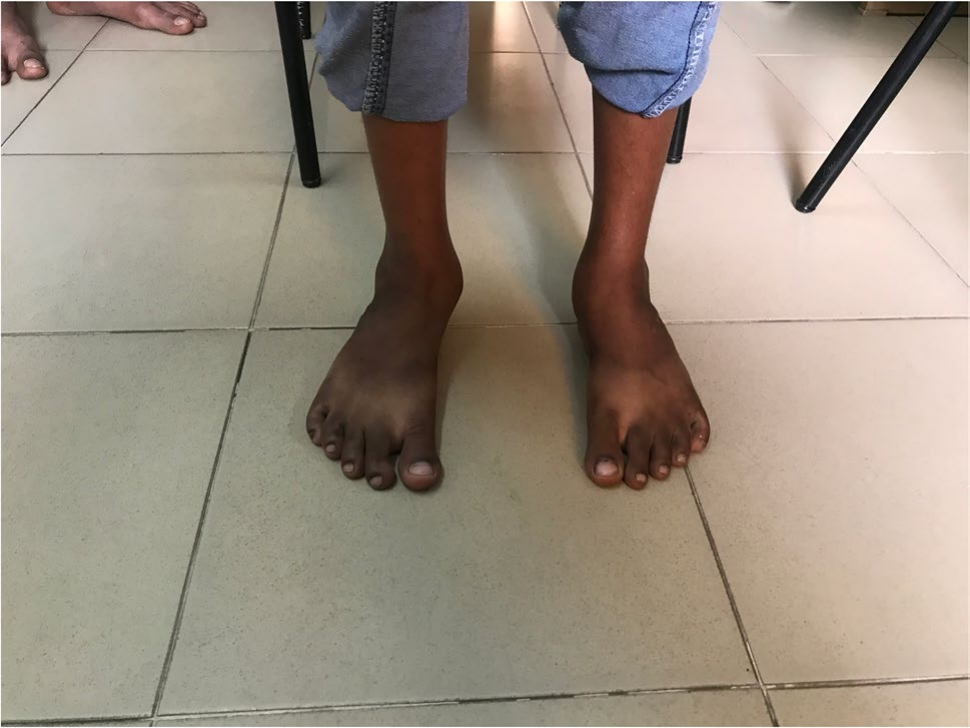

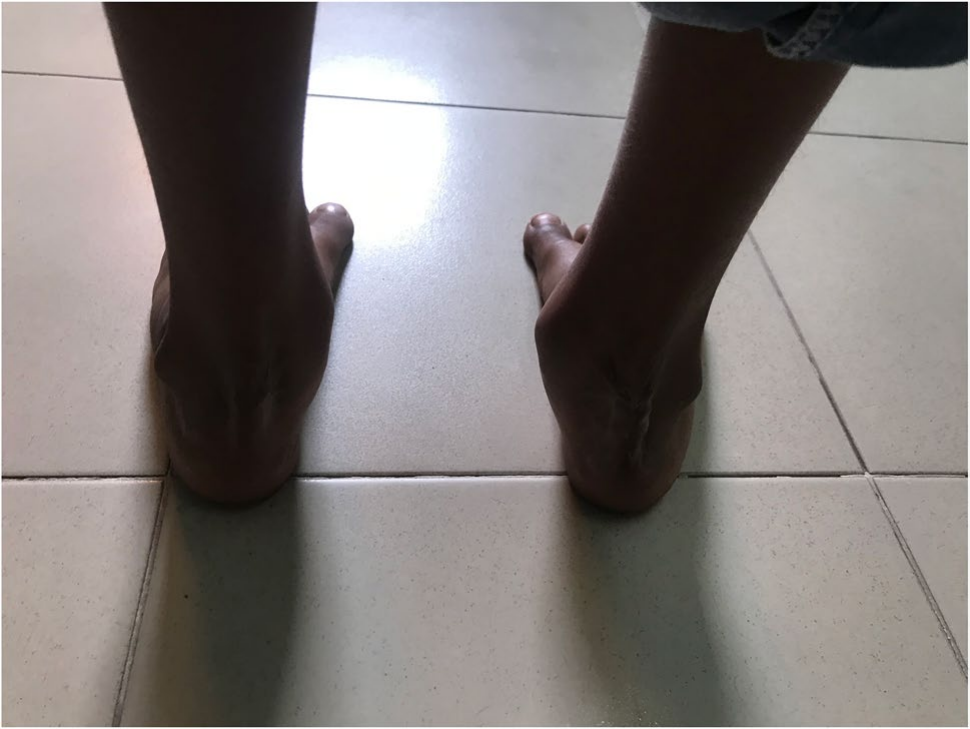

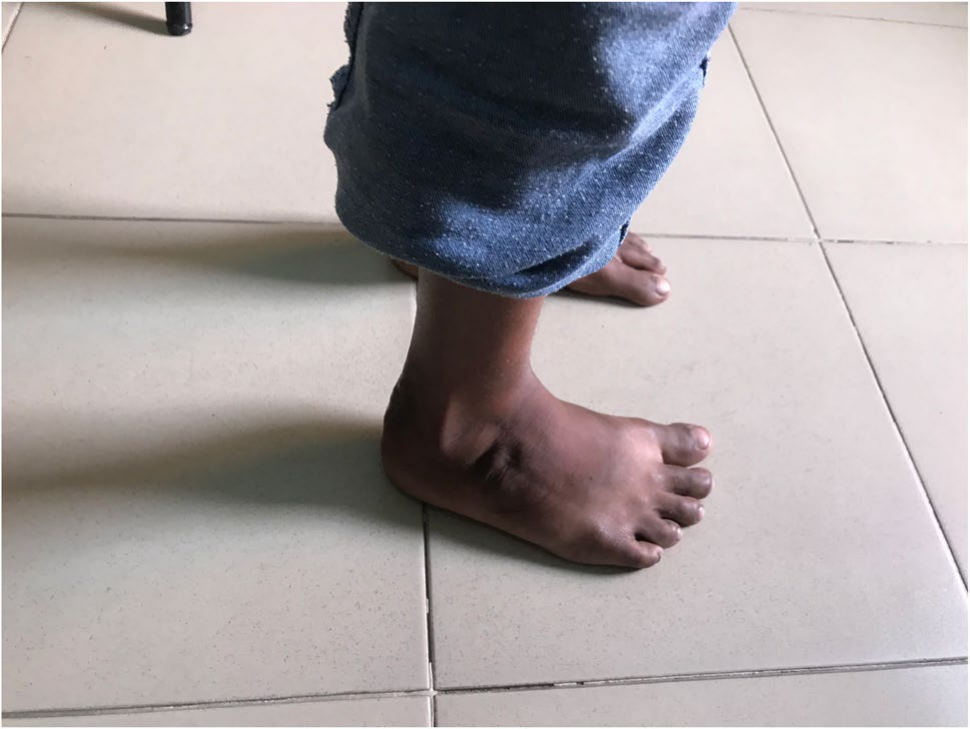

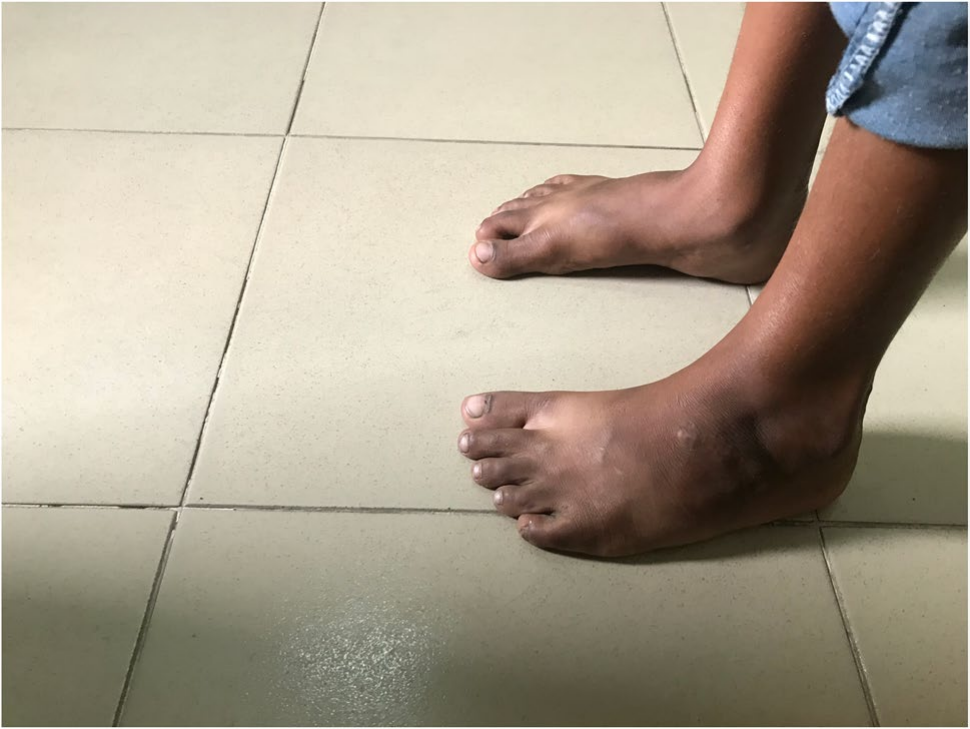

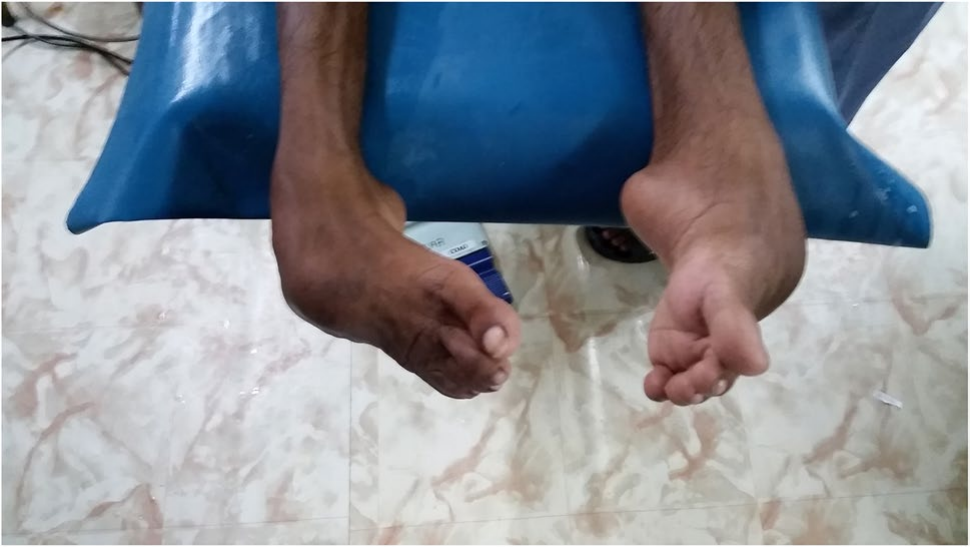

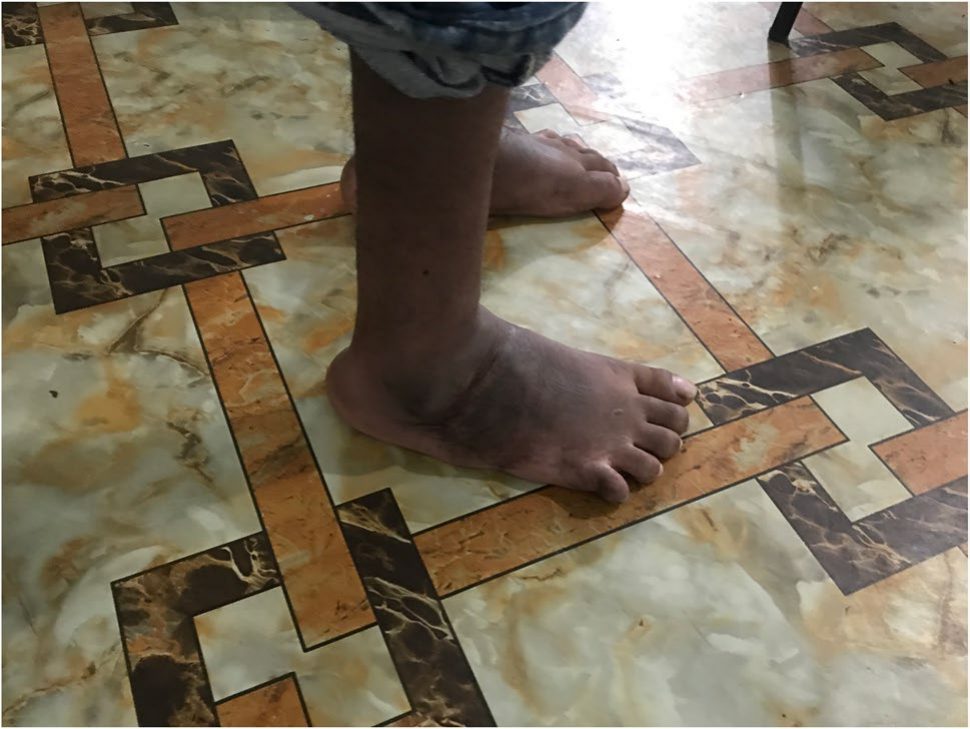
Pre-operative and 2-year post-operative status after bilateral PMR with cuboid osteotomy in a 9-year-old boy

The surgical techniques were adapted to use locally available material, to minimize skin problems in potentially malnourished (protein-deficient) patients and to minimize the risk for pin tract infections. PMRs were performed using a standard Carroll approach [17] as a basis. An additional plantar stab incision is done to ensure a complete disinsertion of the plantar fascia. A release of the distal part of the posterior syndesmosis is done to achieve a better reduction of the talus into the ankle joint. A release of the medial calcaneocuboidal joint capsule is done to achieve a better reduction. A blind release of plantar dermofibrotic adhesions is done, if present. No osteosynthetic material is left behind in the patient post-operatively, including to stabilize the reduced talonavicular joint. Subtraction osteotomies were closed using a thick resorbable thread. Reduction post-operatively is maintained using a correctly molded plaster cast changed every two to three weeks for 15 weeks in total. Photos 1–3 show the post-operative results after two years of a PMR and TATT of the right foot with an excellent Laaveg-Ponseti score. Photos 4–9 show the pre-operative status post-operative results of a bilateral PMR with cuboid osteotomy with an excellent Laaveg-Ponseti score in the left foot and a good one in the right foot.

**Photos. 10-14:**
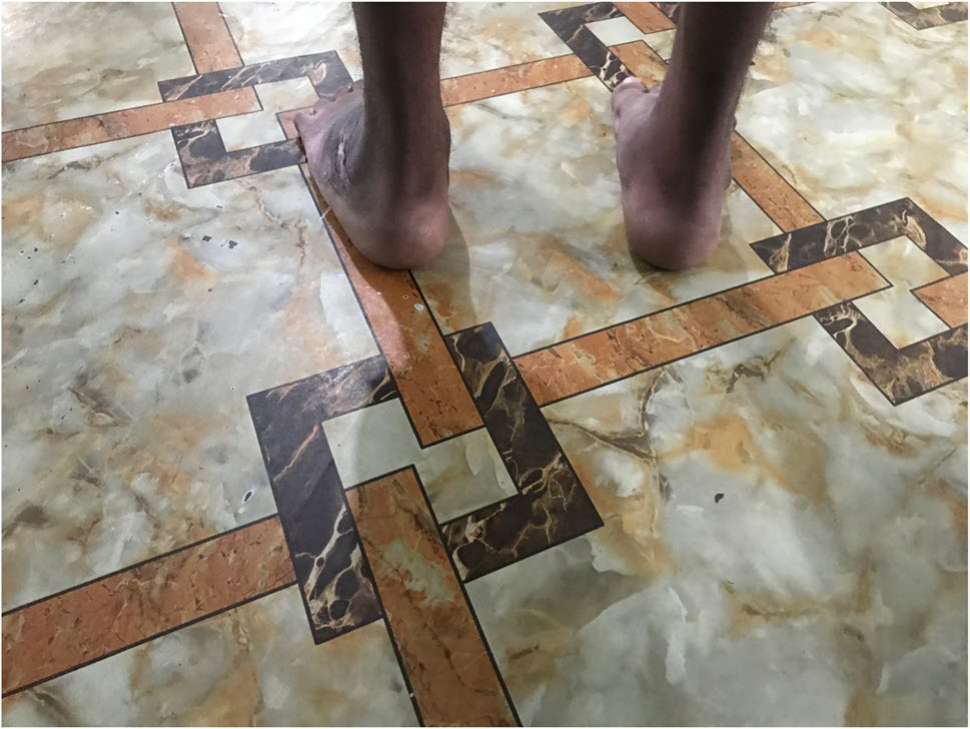

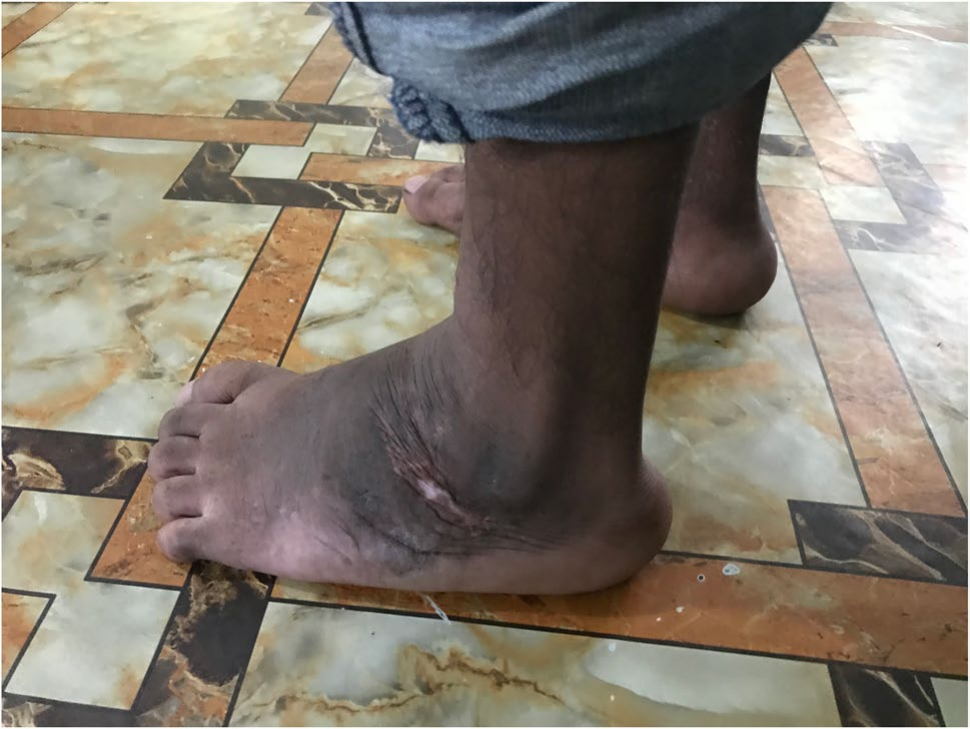

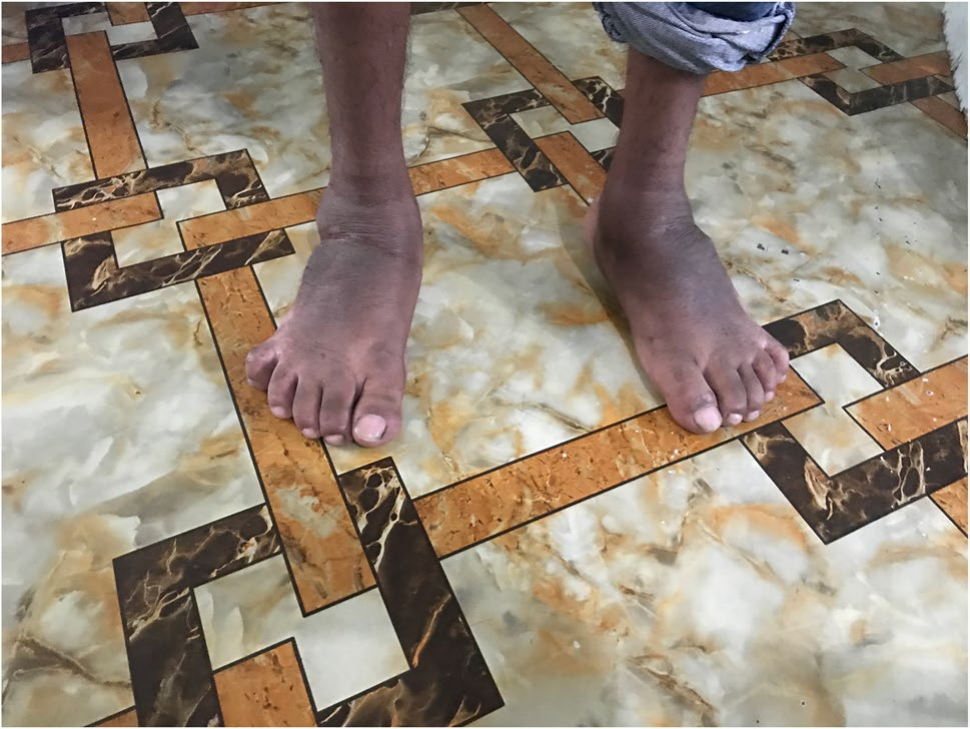
Pre-operative and 2-year post-operative status after bilateral triple arthrodesis in a 21-year-old man

**Photos. 15-17:**
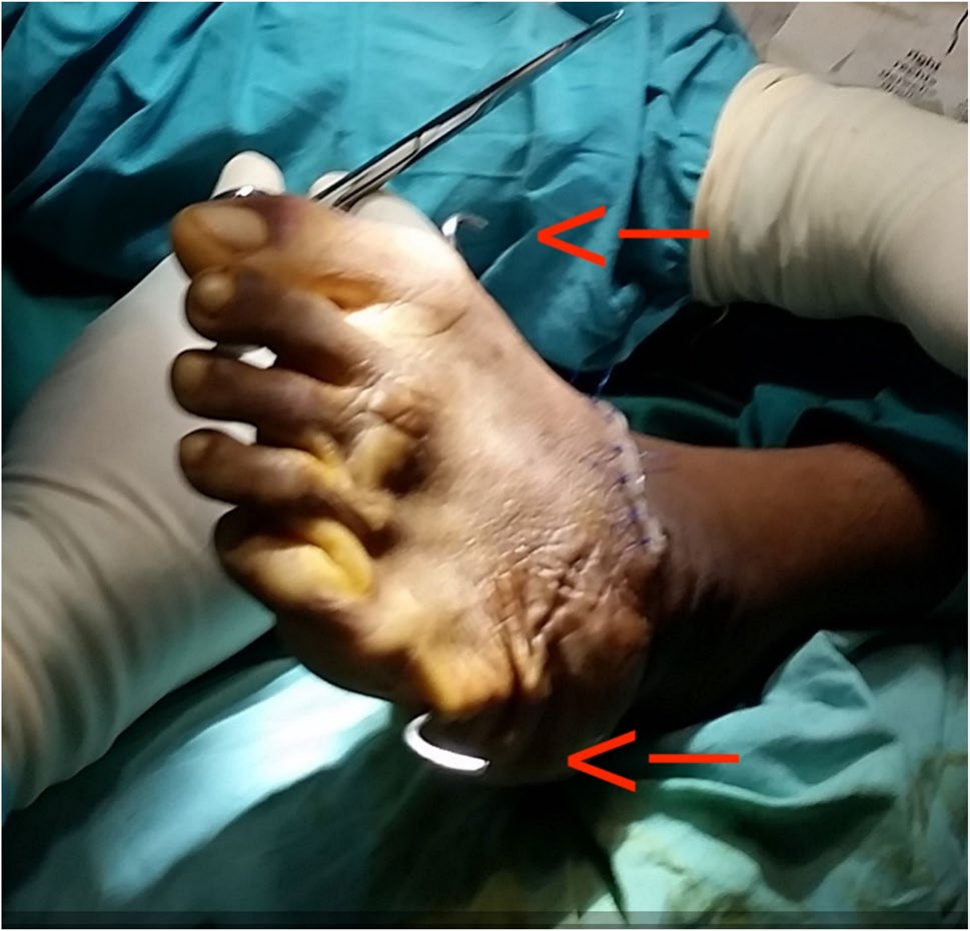

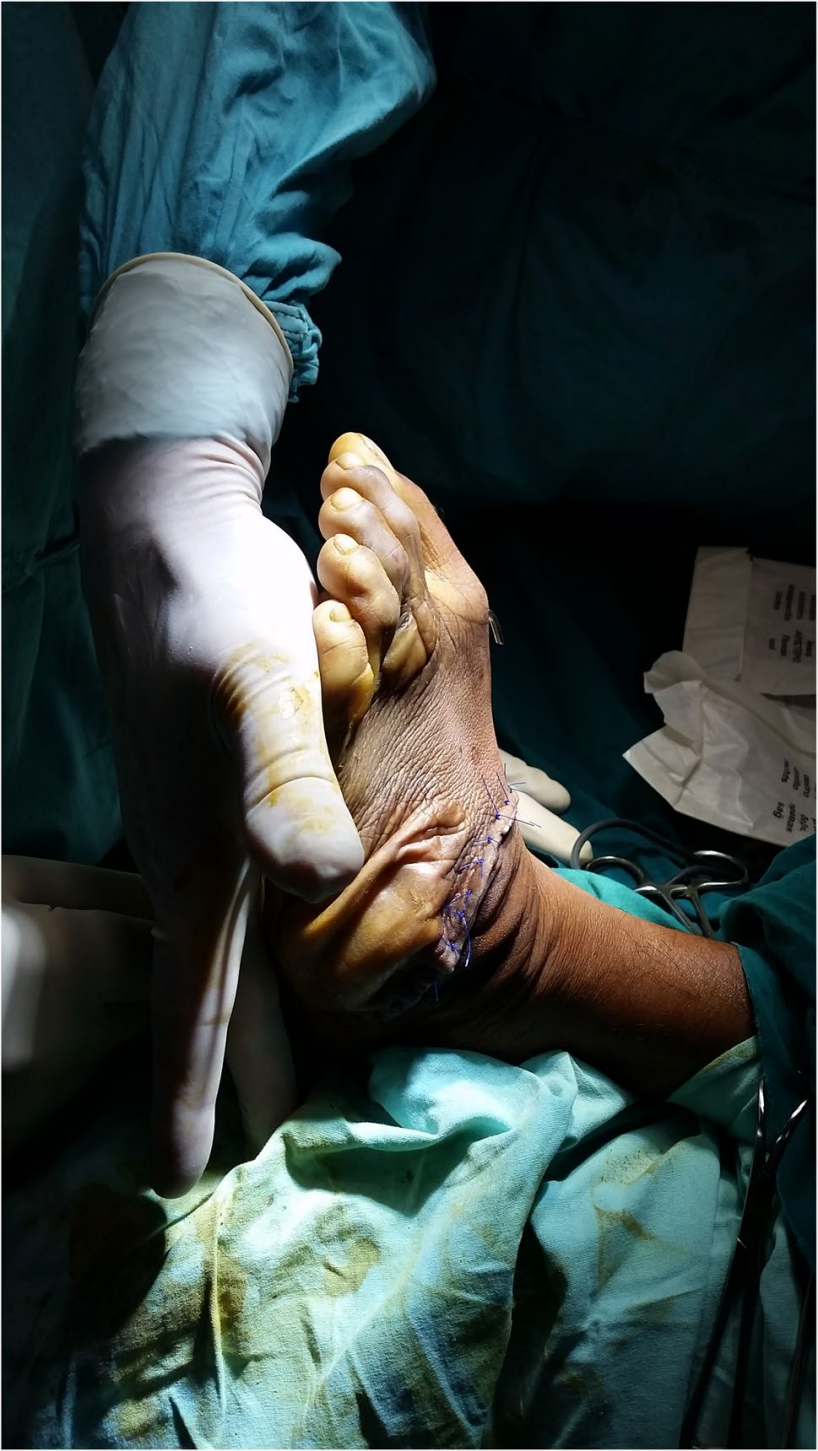

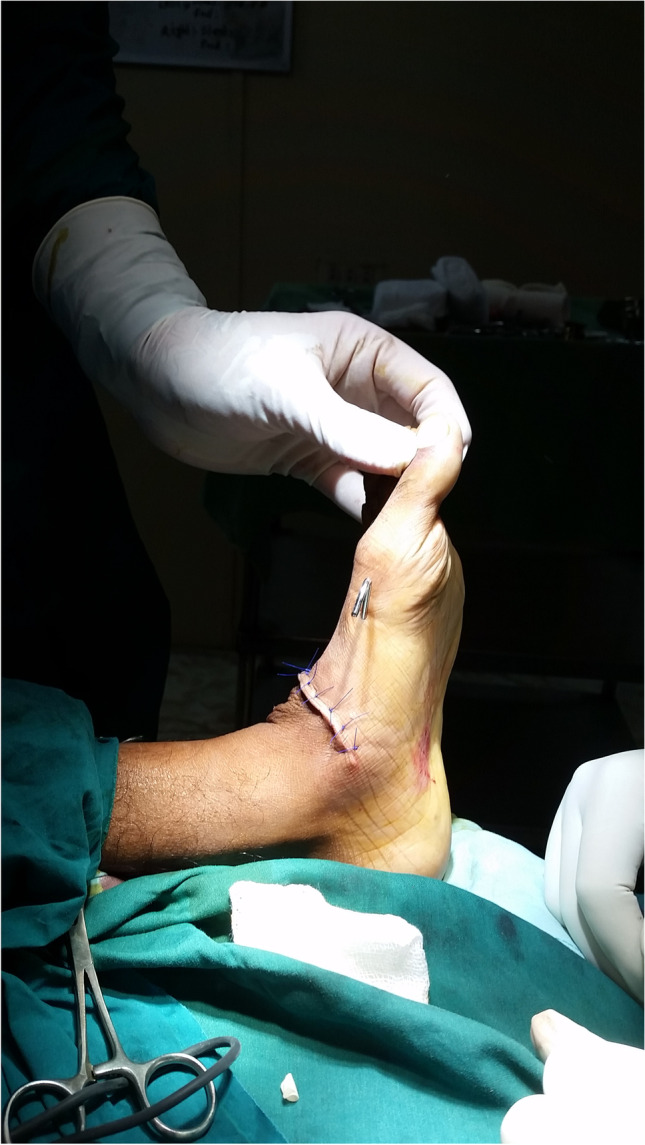
Intra-operative results of triple arthrodesis showing position of dorsal incision and the 2 crossing k-wires (red arrows)

The triple arthrodesis is done according to the Lambrinudi technique [18], using two crossing k-wires in the anteroposterior direction to stabilize the fragments. Reduction post-operatively is maintained using a correctly molded plaster cast for 14 weeks in total. Photos 10–14 show the pre-operative status and post-operative results of a bilateral triple arthrodesis with a bilateral poor Laaveg-Ponseti score. Photos 15–17 show the intra-operative results of the triple arthrodesis, including the position of the dorsal incision and the two crossing k-wires (2 red arrows on photo 15).

A diversion of treatment algorithm was allowed in case of per-operative re-evaluation of the child’s foot by the surgical team. Cuboid subtraction osteotomy was performed as part of the posteromedial release (PMR) when deemed necessary pre-operatively.

All patients were invited for clinical follow-up visits at three months, nine months, 14 months, and 24 months post-operatively. Follow-up at three, six and 14 months was done by a Bangladeshi orthopaedic surgeon from the Impact Foundation, through several house visits in 2018 and 2019. The final follow-up visit was done by a Bangla-Belgian team in December 2019 and included a short social questionnaire.

The “International Clubfoot Study Group (ICFSG) Outcome evaluation score” [19] was used as a basis for the evaluation of the patients’ feet at the first three follow-up visits. For its use in our study, we adapted the ICFSG Outcome evaluation score, in collaboration with the Bangladeshi team on the ground, to an appropriate and feasible evaluation tool to be administered in the context of rural Bangladesh (Supp Fig. 1). This modified version consisted of the morphology evaluation section and the pain evaluation section, culminating in a score between 0 and 15. At the final follow-up visit, all patients were evaluated using the Laaveg-Ponseti score (Supp Fig. 2) [20]. We also asked four questions relevant to evaluate the patients’ social integration at the final follow-up (Supp Fig. 3). These four questions were developed using expert opinion.

For the statistical analysis, patients were divided into three groups: group 1 received a PMR with or without tibialis anterior tendon transfer (TATT) but without additional bony procedures, group 2 received a PMR with at least one of the following bony procedures: cuboid subtraction osteotomy, first metatarsal closing wedge osteotomy or lateral cuneiform closing wedge osteotomy, and group 3 received a triple arthrodesis.

Statistical analysis was done using STATA 16 (StataCorp, Texas, USA). The mean, standard deviation (SD), median, and interquartile range (IQR) are used to describe the baseline characteristics of our study population. Differences in mean ICFSG scores and mean Laaveg-Ponseti scores between groups were analyzed using a Kruskall-Wallis test or a Wilcoxon rank-sum test. A Spearman rank correlation coefficient is calculated to evaluate the impact of age at time of intervention on the Laaveg-Ponseti score. A *p* value of < 0.05 is considered significant.

Institutional review board was obtained at the Institute of Health Economics with registration number FWA00026031 at the University of Dhaka in Bangladesh. Verbal consent was obtained of patients or their legal guardian in the case of minors for their participation in this study, during the last follow-up visit or by phone. Financial support was offered to patients and their parents to cover the costs of transportation for this final visit. No other financial support was offered to patients or their parents.

## Results

In November 2017, the Impact Foundation Bangladesh provided surgical care to 22 patients suffering from 32 clubfeet at their three hospital sites. The average age was 10.0 years with a range of two to 24 years. Ten patients received a bilateral intervention; 12 received a unilateral intervention. Two patients received a different intervention in each foot. The patient baseline characteristics are presented in Table 1. Among the 32 operated clubfeet, 18 were included in group 1, eight in group 2, and six in group 3 (Table 1). All percentages calculated below refer to the amount of operated feet.

**Table 1:** Patient baseline characteristics and follow-up

	Group 1: PMR	Group 2: PMR + bony intervention	Group 3: Triple arthrodesis	*p* value
Operated feet, *n*	18	8	6	
Sex, male(%):female(%)	10(56):8(44)	6(75):2(25)	6(100):0(0)	
Age, mean (SD)	8.2 (4.4)	9.0 (1.7)	22.7 (1.4)*	** < 0.0001**
Intervention subtype, *n*(%)				
PMR	16(89)	-	-	
PMR + TATT	2(11)	-	-	
PMR + cuboid	-	5(63)	-	
PMR + MT1	-	1(12)	-	
PMR + cuboid + MT1 + cuneiform	-	2(25)	-	
Follow-up visit attendance rate, *n*(%)				
3 months	6(33)	1(12)	-	
9 months	9(50)	-	-	
14 months	9(50)	-	-	
2 years	16(89)	5(63)	6(100)	
Modified ICFSG score, median (IQR)				
3 months	4(4–5)	7**	-	0.57
9 months	4(4–6)	-	-	
14 months	5(4–6)	-	-	
Laaveg-Ponseti score at 2 years, mean(SD)	79.1(21.4)	80.8(10.8)	35.3(1.2)	
Excellent (90–100), *n*(%)	7(44)	1(20)	-	
Good (80–89), *n*(%)	4(25)	1(20)	-	
Fair (70–79), *n*(%)	2(12)	2(40)	-	
Poor (< 70), *n*(%)	3(19)	1(20)	6(100)	
2-year follow-up (Laaveg-Ponseti score), median (IQR)	85.5(71–93.5)	77(74–89)	36(35–36)	***0.0038***
Post hoc subgroup analysis with Bonferroni correction				
Group 1 vs 2				1.0000
Group 2 vs 3				***0.0234***
Group 1 vs 3				***0.0016***

^*^Group 3 is significantly different from group 1 and 2

^**^No interquartile range available for only one observation

Cuboid, cuboid subtraction osteotomy

MT1, first metatarsal closing wedge osteotomy

Cuneiform, lateral cuneiform closing wedge osteotomy

All 22 patients who received surgery have been contacted for follow-up visits. Three patients were lost to follow-up. On the contrary, 19 patients (13 were male and 6 were female) suffering from 29 clubfeet (90.6%) attended at least one follow-up visit. Follow-up visit attendance was very good at the two year follow-up visit (84%) but overall irregular during the intermediate follow-up (Table 1). The attendance rates for group 1 range between 33 and 89%. In group 2, 1 patient attended a follow-up visit at three months and 63% attended the final two year follow-up visit. All patients in group 3 attended the two year follow-up visit but none presented before.

The modified ICFSG score could only be compared for the three month follow-up visit, and only between group 1 and 2. The difference is not statistically significant.

At two year follow-up, 81% of the patients in group 1 had a Laaveg-Ponseti score that was excellent, good, or fair, compared to 80% in group 2 and 0% in group 3 (Table 1). The difference in Laaveg-Ponseti median scores at the two year follow-up visit is statistically significant between the 3 groups with the higher median in group 1 (85.5%). A post hoc subgroup analysis with Dunn’s test shows that group 3 has a significantly lower score compared to group 1 and 2. No significant difference could be found between group 1 and 2. The patient’s age at the time of intervention is significantly correlated with the Laaveg-Ponseti score at two year follow-up (*p* value < 0.0001). The younger the child is at the time of the surgical intervention, the higher the Laaveg-Ponseti score is at two year follow-up.

The social questionnaire at two year follow-up showed that all 19 evaluated patients were enrolled in school or employed and were able to wear normal shoes. Only 1 patient (bilateral triple arthrodesis) was not able to squat and experienced an overall decrease in quality-of-life compared to pre-operatively.

## Discussion

The aim of our study was to retrospectively investigate the morphological, functional, and social outcomes in patients with neglected clubfoot in rural Bangladesh, after receiving surgical treatment. We found that children undergoing a PMR with or without TATT or additional bony interventions obtained good results. Triple arthrodesis in our cohort gave very poor results.

Comparing outcomes after surgical treatment for neglected clubfoot between studies from LMICs remains very difficult, because of a plethora of inclusion criteria, surgical techniques, and evaluation criteria used. Comparing outcomes between Ponseti treatment and surgical care in LMICs remains equally difficult [21]. However, studies from high-income countries clearly show that long-term outcome of surgically treated clubfoot is poorer compared to feet treated with the conservative Ponseti treatment protocol, in terms of pain and foot morphology [13, 22–24]. Studies about the effectiveness of Ponseti treatment in children above walking age in LMICs show promising results. Extensive soft tissue releases have been avoided in 66–92% of cases above 1 year of age [8, 25, 26]. The Ponseti treatment remains the golden standard for clubfoot treatment in low-, middle-, and high-income countries [7–9]. As such, we strongly believe that Ponseti casting should remain the treatment of first choice, including for older children, when available and appropriate.

Our proposed treatment algorithm seems to be well in line with other surgical algorithms used in LMICs. We consider it a pragmatic approach that takes into account the reality that the Ponseti treatment is not always available or feasible, while the burden of untreated neglected clubfoot is enormous. All patients in our cohort were either enrolled in school or employed. This change in social status after surgery will have far-fetching positive impacts on their own lives and on their communities and should not be underestimated.

Previous studies in LMICs have shown comparable results for the outcome after PMR surgery [12, 27, 28]. Faldini et al. have followed two cohorts in Eritrea (2- to 5-year-old) and Tanzania (6- to 9-year-old) who underwent a soft tissue intervention combined with a cuboid subtraction osteotomy. Patients had good to excellent outcome based on the Laaveg-Ponseti score in respectively 91% in Eritrea after an average follow-up time of two years [28] and in 79% of the cases in Tanzania after an average follow-up time of five years [12]. Hoque et al. operated on two cohorts between the ages of six months and 16 years with PMR without additional bony interventions. They achieved predominantly good and excellent as well [27], based on the Turco score that cannot be directly compared with the outcomes evaluated with the Laaveg-Ponseti score.

There is a certain subgroup of patients with residual deformities (bean-shaped foot) after PMR that requires additional bony interventions for neglected clubfoot [12, 29]. However, as stated by several authors with experience with neglected clubfoot in LMIC before, this decision should be bas5ed on a thorough clinical evaluation and not on the age at intervention [2, 12, 29]. Our data reconfirms this policy and reconfirms the previous data of Hoque et al. that excellent results can be obtained with an isolated PMR regardless of age [27].

The ideal age for surgical treatment of neglected clubfoot remains controversial [30]. El-Tayeby et al. found no correlation between age at intervention and outcome in their cohort of 28 children and adolescents operated upon with PMR in Egypt [31]. The outcome evaluation score used was the modified Abrams’ criteria, as such results are difficult to compare between our study and theirs. In our study, there was a clear and strong correlation between age at intervention and the outcome at two year follow-up, which would support the advice to operate neglected clubfoot as early as possible. These findings are in line with the findings of Hoque et al. who found that the best results with PMR were obtained in children below the age of three years, and Eidelman et al. based on their extensive experience [27, 32].

Reports about short-term outcomes of triple arthrodesis for neglected clubfoot in LMICs remain anecdotal [2, 27]. When looking into the older literature from high-income countries, results appear similar to ours. Herold et al. and Angus et al. both considered triple arthrodesis a salvage procedure for neglected clubfoot and reported predominantly poor functional results in their respective patient cohorts [33, 34].

Our study has severe limitations because of the large heterogeneity among our patients in terms of age at intervention and type of surgical intervention received. The lack of a validated pre-operative scoring makes it difficult to compare our results with other studies. The small sample size for our triple arthrodesis sub-cohort in combination with the lack of reliable comparative data from the literature, makes it very difficult to interpret our data and its generalizability beyond our setting in rural Bangladesh. The follow-up period of only two years makes it impossible to assess the impact of puberty on the operated feet and possible subsequent changes in functionality and morphology.

PMR as a treatment for neglected clubfoot in LMICs has consistently showed good to excellent results in children and adolescents. We believe PMR can help alleviate the burden of neglected clubfoot in LMICs in a safe way with acceptable outcomes. Especially in regions where Ponseti treatment is unavailable or considered inappropriate for a specific patient, PMR can complement treatment options. Surgical treatment should be initiated as early as possible, if Ponseti treatment is unavailable, because of a clear influence of age on the final result. The final choice of intervention should be made by the operating surgeon based on pre- and per-operative findings and evaluation. Further research is needed to investigate the possible role triple arthrodesis can play in alleviating the burden of neglected clubfoot among young adults in LMICs.

## Supplementary Information

Below is the link to the electronic supplementary material.

Supplementary Fig. 1 Modified ICFSG Outcome evaluation score, from Bensahel et al. (19) (PNG 194 KB)

Supplementary Fig. 2 Laaveg-Ponseti score, from Laaveg et al. (20) (PNG 212 KB)

Supplementary Fig. 3 Questionnaire for evaluation of social integration. (PNG 75 KB)
