# Supplementary figures and images for: Evaluation of a surgical treatment algorithm for neglected clubfoot in low-resource settings
Source: Int Orthop. 2021 Jun 19;46(1):61–70. doi: 10.1007/s00264-021-05058-6 (PMC8752568; doi:10.1007/s00264-021-05058-6)

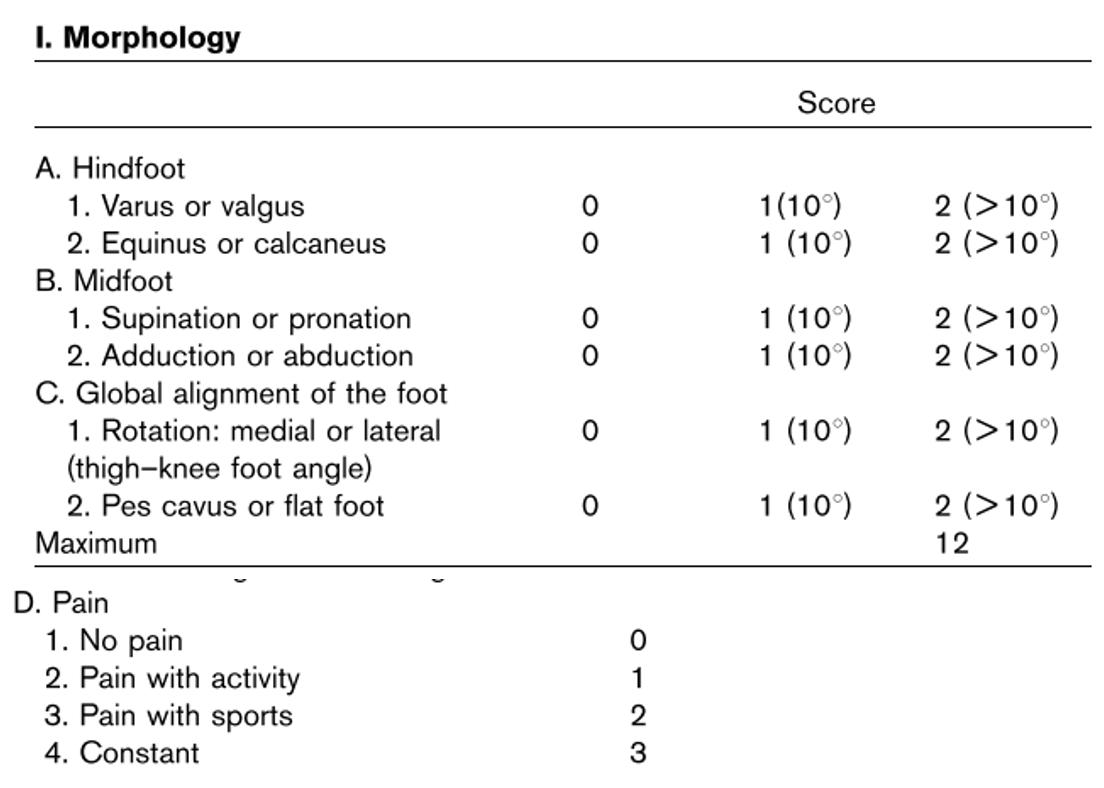

Supplement: Supplementary file 1 — Supplementary Fig. 1 Modified ICFSG Outcome evaluation score, from Bensahel et al. (19) (PNG 194 KB) [file 264_2021_5058_MOESM1_ESM.png]

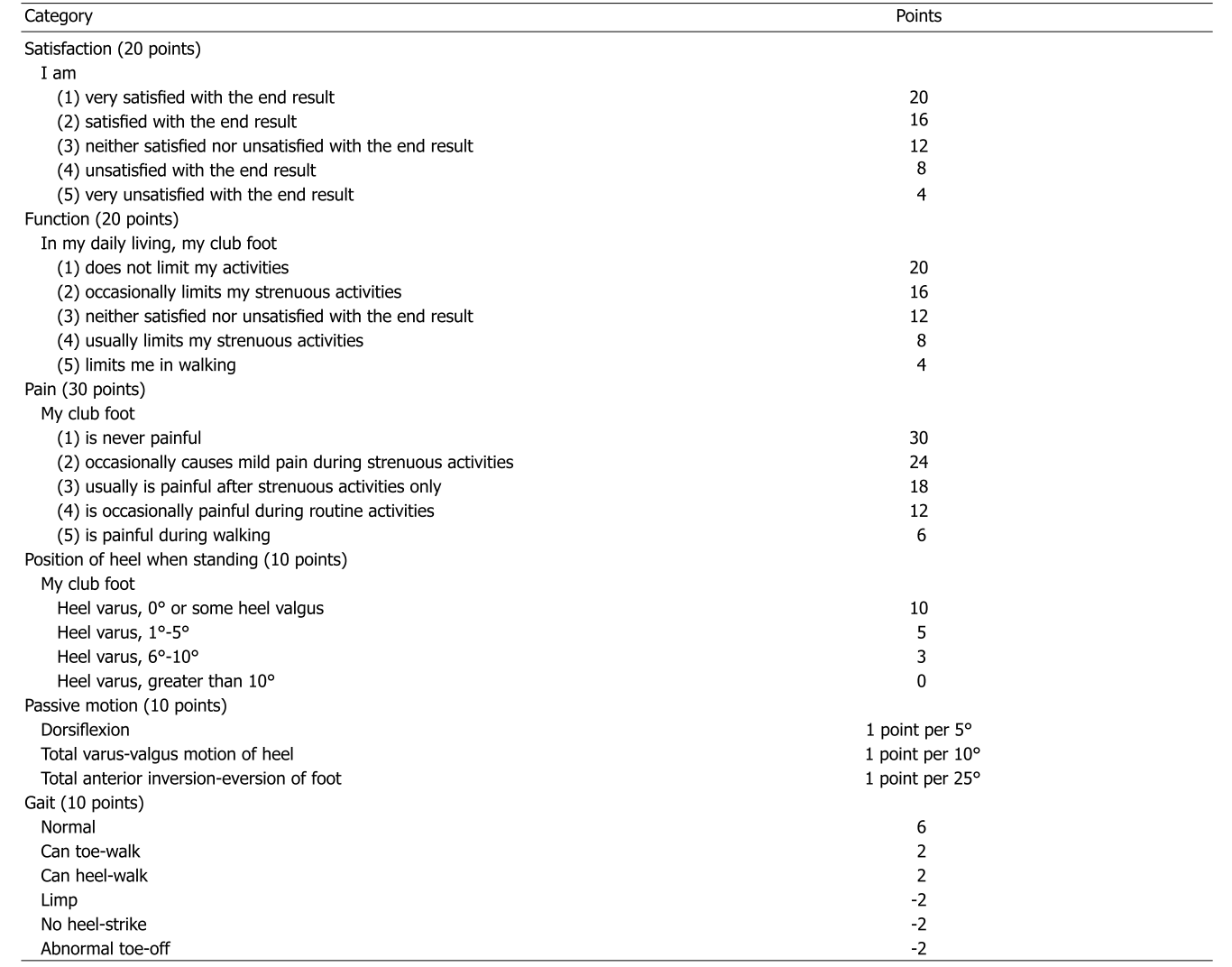

Supplement: Supplementary file 2 — Supplementary Fig. 2 Laaveg-Ponseti score, from Laaveg et al. (20) (PNG 212 KB) [file 264_2021_5058_MOESM2_ESM.png]

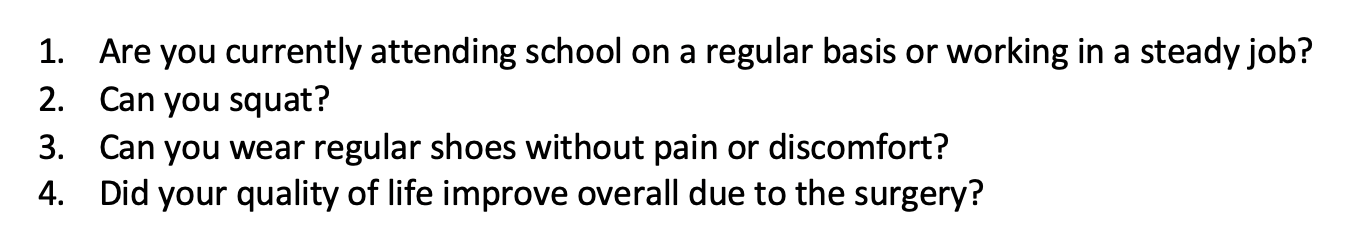

Supplement: Supplementary file 3 — Supplementary Fig. 3 Questionnaire for evaluation of social integration. (PNG 75 KB) [file 264_2021_5058_MOESM3_ESM.png]
